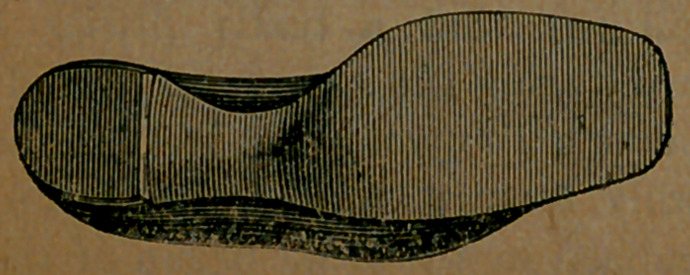# Shall We Possess Our Soles in Comfort?

**Published:** 1875-05

**Authors:** 


### SHALL WE POSSESS OUR SOLES IN COMFORT?

THIS is the question, a question as important, perhaps, as any with which we have to deal. For, investigate as we may, we can never fully comprehend, in all their alarming magnitude, the evils suffered by the race from distorted and abused feet.

If, then, we can Possess our Soles in Comfort we shall be doing a grand work, not only in our own behalf, but in behalf of those who are to succeed us, and who will be symmetrical or deformed, beautiful or distorted, noble or ignoble, according as they inherit from us wholesome or vicious tendencies.

For it is a fact, affirmed and demonstrated, that children necessarily inherit the physical and mental tendencies of their parents; and physiologists assert that the power of transmitting impressions of body and brain, is wonderfully vivified during the period of maternity. If this be true—and we know it cannot be disproved—who shall say that a badly fitting shoe, worn during the supremest period of a woman's life, may not have induced a bodily torture which the brain has localized in the same organ of the offspring, inducing thereby club-foot and permanent deformity ?

There is a growing tendency among civilized peoples, to foot-deformity. Some of the older nations of Europe have a greater tendency in this direction, than have the people of the United States, because the deforming processes have continued for a longer period. If we examine the foot of almost any native of the Prussian Empire or of any of the German States, we shall find enormous bulging joints at the base of the great toe, and hideous deformity. We shall see, too, that this deformity has induced an awkward, shambling gait, or sidewise dragging of the feet, and a total lack of ease and grace and dignity of personal bearing˙ It is only a question of time for Americans to become as awkward and unelastic as are these victims of the long-continued oppressions of shoemakers.

Happily, relief is offered to such as see fit to accept it. It is radical relief, too, and not a mere theory. It takes the form of a block of wood representing a well-formed human foot. Upon this block of wood, leather is drawn, and neatly fastened together. When this is completed, the block of wood is withdrawn and the foot is put in its place. The sensation is a pleasant one, because the foot is not injured ; it is not unequally compressed.

Here may be seen the bottom of one of these properly-formed leather coverings. It will be observed that it does not "run over" at any point. Its effect is neat, and all that can be desired.

Take now a similar view of a boot worn for about the same length of time, by the same person. It was made on the usual plan and is no worse than the average boot after being worn a month or two. Of course its construction was all wrong ; but so is the construction of nearly all the boots and shoes made in the world. This was made on a block of wood, too, but it was not a block shaped like a healthy human foot ; it was not a block shaped like anything known to human beings, except its fellows. Millions of such blocks of wood are made each year in the United States ; and until Joel McComber applied his inventive skill to the work, there was no improvement in them. Now, however, things are changing, and the day is not very far off, when McComber's "Lasts" will have to be used instead of the barbarous blocks now so largely employed.

When we began to write, we meant to speak of our excellent friend, Mr. F. Edwards, who keeps a great boot and shoe factory and store at 166 and 168 Atlantic avenue, Brooklyn, and who uses only McComber's lasts. In fact, he alone, of all the dealers in Brooklyn, has the right to use this invention, a right which he has bought from the inventor. He is doing a very large business, as he should, and we hope all our Long Island friends will apply to him whenever they reach the decision that in future they will Possess their Soles in Comfort.

---

There is nothing like system in exercise. If you are weak, begin gradually. Dress up and go out if you only walk ten rods. The next day walk eleven, and so on till you are strong. You will be surprised to discover how surely strength comes.

If you would be beautiful avoid late hours.

### THINGS TO BE REMEMBERED.

REMEMBER always that PAIN is the warning cry of a faithful sentinel on the outpost. The words which this sleepless watchman utters are, "Take care! Disease is at hand." The enemy thus announced is the punishment following a violation of the laws of nature, and this enemy can only be evaded by restoring natural conditions.

Remember, also, that nature never slumbers nor fails in duty, but strives with unerring, active intelligence to prevent disease, or to cure it when it cannot be prevented.

When the measures and processes of the physician are in harmony with the natural intention, disease may be cured; when they are adverse in application, the patient dies, or if he recovers, it is in spite of intermeddling.

A great French philosopher once said: Nature fights with disease a battle to the death; a blind man armed with a club—called a physician—comes in to make peace between them. Failing in that, he lays about him with his club. If he happens to hit disease he kills disease; if he hits nature he kills nature.

Let us see if we cannot do something to assist and facilitate the operations of nature in her constant warfare with the diseases and accidents of life. Let us try to rid ourselves of the blindness which the philosopher attributed to the Doctor. If there be a ray of light to be found, with which the all-important secrets of human well-being can be illumined, let us seek for and find it.

Let us be *reasonable;* by which we mean, not merely *able to reason*, but able to apply the results of our reasoning.

---

Don't ride in winter; to walk will give you rich blood; to ride will perhaps shorten your days.

CONSUMPTIVE PEOPLE are, as a rule brilliant people. It is sometimes simply wonderful to observe the mental power and activity of those who are in the early stages of serious lung disease. The pulse is rapid, the heat great, the eyes bright and the brain unnaturally excited. Then it is that the poet composes verses with readiness, and imagination and fancy are vivid and alert. This is obviously due to the nerve-communication between the lungs and the brain, by which the excitement of the one is telegraphed to the other, with the effect of exalting its powers. The history of literary men who have suffered from gout, shows that in the presence of that painful disease the power of the brain is greatly increased. This is no doubt due to the excess of nitrogen in the system. Nitrogen always exists in large quantity in the blood of persons afflicted with the gout; and it is well known that nitrogen is looked upon by physiological chemists as the best brain-food. It has been found to give strength to the will and new aptitude for mental work.

---

So great is the demand for the spurious literature sent out week after week by the "story papers" that steam, with all its giant capabilities, is scarcely able to meet the demand. A taste had at first to be created before the cry was raised for a large supply to satiate the public appetite. Men were found willing to spend fortunes in creating this vitiated taste, well knowing that they should eventually succeed in dragging the pure and youthful to the level that would yield them large returns.

---

Corns and sore feet are more rapidly relieved by the use of soap containing carbolic acid than by any other means.

POISONOUS SNAKES.—It is a remarkable fact that the virus of a poisonous snake is comparatively harmless when taken into the stomach. The most venomous snakes seem to possess a perfect immunity from the poison of their own species, not being able to poison themselves or each other. It is found that carbolic acid injected under the cuticle, speedily destroys most of the venomous snakes. "Instinct," accordingly, induces the greatest repugnance in these reptiles to this acid. Poisonous snakes quickly learn the spot where the solution has been sprinkled, and will not bite an animal who has been smeared with it. Advantage may be taken of this fact by persons travelling in infested regions. The clothing may be sprinkled with a weak solution of this acid, and the exposed portions of horses and other animals may be washed with it.

---

WHEN we look at the magnitude and variety of the evils of this age we find it hard to point to one so terribly disastrous in its effects as the spread of indecent and immoral literature. We have laws for the suppression of obscene publications, but there are no laws to prevent the wide circulation of that class of literature which is eagerly sought after by the youth of both sexes, and is known to be both deadly and destructive in its tendency and calculated to inflict irreparable injury on the minds and morals of all those who indulge in its perusal. This literature comes in the innocent guise of "story papers," and is as attractive as wine, and a thousand times more dangerous. Its intoxication lasts and kills. Better set a whisky-jug before your son or daughter, than supply them with the weekly trash which all the great cities send forth to do its poisonous work.

There can be no better food than beans. They are full of nourishment and warmth. They are probably the cheapest food used in this country. But to be really valuable they must be well cooked. As they are usually brought on the table they are bad for the stomach and bowels, because they are not cooked enough. Boil them till you can't discover the shape of a bean in a big dish full, and they are then just ready to eat. The old distitch which we here quote, simply means that you can't cook beans too much:

Bean porridge hot and bean porridge cold,
And bean porridge in the pot nine days old.

---

LOCKJAW, or tetanus, has been deemed impossible to cure, but it now appears that its spasms and contractions are completely managed by the administrations of nitrate of amyl, which has the power of relaxing every fibre of the individual, the blood-vessels and muscle alike. Even the spasms attending the poison by strichnine are overcome, and its value as an anti-spasmodic is especially demonstrated in that most terrible malady called augina pectoris, in which the paroxysms have been lessened and even removed.

---

Snoring in sleep is a disease rather than a habit. It indicates that the snorer breathes through the mouth and not the nose. People who snore habitually are short-lived, becaase the air inhaled is not strained or filtered before passing into the lungs.

---

Thousands of diseases may be cured by simply rubbing, kneading and percussing the body.

---

If your breath is foul your blood will be poor, because you contaminate your blood with every breath you draw.

## Figures and Tables

**Figure f1:**
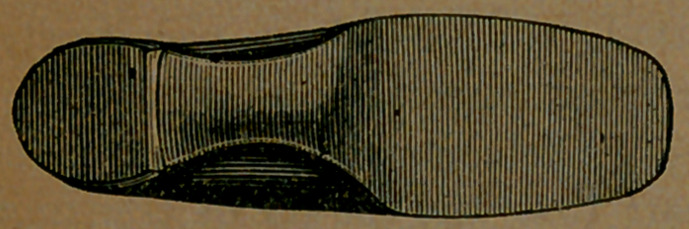


**Figure f2:**